# A proof-of-concept assay for quantitative and optical assessment of drug-induced toxicity in renal organoids

**DOI:** 10.1038/s41598-023-33110-5

**Published:** 2023-04-15

**Authors:** Jasmin Dilz, Isabel Auge, Kathrin Groeneveld, Stefanie Reuter, Ralf Mrowka

**Affiliations:** 1 Department of Internal Medicine III, Experimental Nephrology, Jena University Hospital, Nonnenplan 4, 07745 Jena, Germany; 2 ThIMEDOP, Jena University Hospital, Nonnenplan 4, 07745 Jena, Germany

**Keywords:** Cell biology, Stem cells, Molecular medicine

## Abstract

Kidneys are complex organs, and reproducing their function and physiology in a laboratory setting remains difficult. During drug development, potential compounds may exhibit unexpected nephrotoxic effects, which imposes a significant financial burden on pharmaceutical companies. As a result, there is an ongoing need for more accurate model systems. The use of renal organoids to simulate responses to nephrotoxic insults has the potential to bridge the gap between preclinical drug efficacy studies in cell cultures and animal models, and the stages of clinical trials in humans. Here we established an accessible fluorescent whole-mount approach for nuclear and membrane staining to first provide an overview of the organoid histology. Furthermore, we investigated the potential of renal organoids to model responses to drug toxicity. For this purpose, organoids were treated with the chemotherapeutic agent doxorubicin for 48 h. When cell viability was assessed biochemically, the organoids demonstrated a significant, dose-dependent decline in response to the treatment. Confocal microscopy revealed visible tubular disintegration and a loss of cellular boundaries at high drug concentrations. This observation was further reinforced by a dose-dependent decrease of the nuclear area in the analyzed images. In contrast to other approaches, in this study, we provide a straightforward experimental framework for drug toxicity assessment in renal organoids that may be used in early research stages to assist screen for potential adverse effects of compounds.

## Introduction

On average, the Food and Drug Administration approves one out of ten potential drug candidates in clinical trials^1,2^. During drug development potential compounds often fail to meet clinical safety standards due to unexplained toxicity, thus causing an immense financial loss for pharmaceutical companies^2,3^. As a result, there is a constant need for more accurate model systems. In the last 10 years, researchers could observe a rapid rise of a new in vitro model system of 3D multicellular organ-like complexes known as organoids^4^. This method takes advantage of the ability of stem cells to self-organize into 3D structures^5^. Organoids closely mimic the structural and functional characteristics of real organs and can thus exhibit near-physiological responses^6^. Furthermore, they display cellular heterogeneity and facilitate organotypic intercellular communication similar to in vivo conditions, making them an excellent model system^7^. Established organoid models have already aided in the development of new therapeutics for diseases such as cystic fibrosis, Crohn’s disease, and ulcerative colitis^8,9^. The generation of patient-derived organoids (PDO) from patient-derived induced pluripotent stem cells (iPSCs) opened new possibilities for personalized drug screening and treatment. Remarkably, PDOs are increasingly being used to identify new anti-cancer agents or to recapitulate patient-specific drug responses^10–12^. According to ClinicalTrials.gov, 152 clinical trials currently employ organoids as model systems, most of which are cancer-related^13,14^.

The adult metanephric kidney develops from the mesodermal germ layer through a bidirectional induction between the ureteric bud (UB) and the metanephric mesenchyme (MM), the kidney precursor lineages^15,16^. Through constant reciprocal interaction and self-organization, the UB eventually produces collecting ducts and ureters, whereas the MM forms the mature nephron^15,17^. The process of the formation of hiPSC-derived renal organoids mimics these signaling events by supplementing the culture media with small molecules, like the WNT activator CHIR99021, which induce the differentiation of human iPSCs (hiPSCs) to mesodermal cells and direct cell development towards the renal lineage^18^. In general, renal organoids are made up of segmentally patterned nephron-like structures, stromal cells, and endothelial cells that are highly similar to the human fetal kidney^19,20^.

Drug-induced kidney injury is a common side effect during the course of treatment. Although 2D cell cultures and animal models helped advance the research in this field, they appear to be not as predictive of human responses^21^. It is estimated that around 20% of compounds in third-phase clinical trials fail because they exhibit unexpected adverse nephrotoxic effects^22,23^. These events are also known to cause structural renal damage and functional impairment, a syndrome known as acute kidney injury (AKI)^19,24,25^. AKI does not only increase the risk for chronic kidney disease but is also attributed to high morbidity, especially in patients with preexisting conditions^26,27^.

The use of renal organoids to model responses to nephrotoxic insults has the potential to bridge the translational gap between preclinical drug efficacy studies in cell cultures and animal models, and the stages of clinical trials in humans^28^. The establishment of personalized organoid biobanks from PDOs can bring valuable additions to drug testing and sensitivity screening, potentially lowering the risk of AKI development due to drug-induced toxicity^29^. Although more research progress on kidney organoid technology is needed to recreate physiologically responding kidney models in vitro, the studies conducted thus far show great promise^30^.

Doxorubicin (DOX) is a chemotherapeutic agent used to treat various types of cancer^31,32^. But aside from proliferative cancer cells, DOX also affects healthy cell populations and can cause severe organ damage, even at therapeutic doses^33,34^. The cytotoxicity of this quinone-containing anthracycline is hypothesized to be attributed to two central mechanisms: DNA alkylation causing transcriptional interference, and the increased formation of reactive oxygen species (ROS), with the latter being most likely to cause adverse effects during treatment^35–38^. The resulting imbalance between the presence of radicals like ROS and radical scavengers (oxidative stress) can disrupt the mitochondrial respiratory chain and ultimately cause cell apoptosis^39–41^. A recent study by Nagai et al*.* showed that DOX-induced oxidative stress was associated with renal impairment in DOX-treated rats. It caused microscopically visible deterioration of kidney tissue histology, loss of cellular boundaries, and bleeding in the rodent model^42^.

Other studies also revealed histological features similar to focal glomerular sclerosis in humans, such as podocyte injury and chronic tubulointerstitial fibrosis following DOX treatment^43,44^. Recently, Hale and colleagues developed a human 3D glomeruli organoid model in order to study DOX-induced renal insult. They genetically tagged podocytes with a fluorescent protein and measured the fluorescence signal over the course of DOX treatment. As a result of the DOX-induced toxicity, they observed a concentration-dependent loss of signal, along with fragmentation and destruction of glomeruli^6,45^. Kumar et al. used confocal analysis of TUNEL-positive cells to show that 2.5 and 5 µg/mL DOX-concentrations had an overall toxic effect on kidney organoids in addition to causing podocyte injury. They further observed a dose-dependent reduction in gene expression of renal marker genes. Interestingly, this not only affected podocyte genes, but also markers of other tubule segments^7^.

## Results

### Organoid characterization

**Figure 1:**
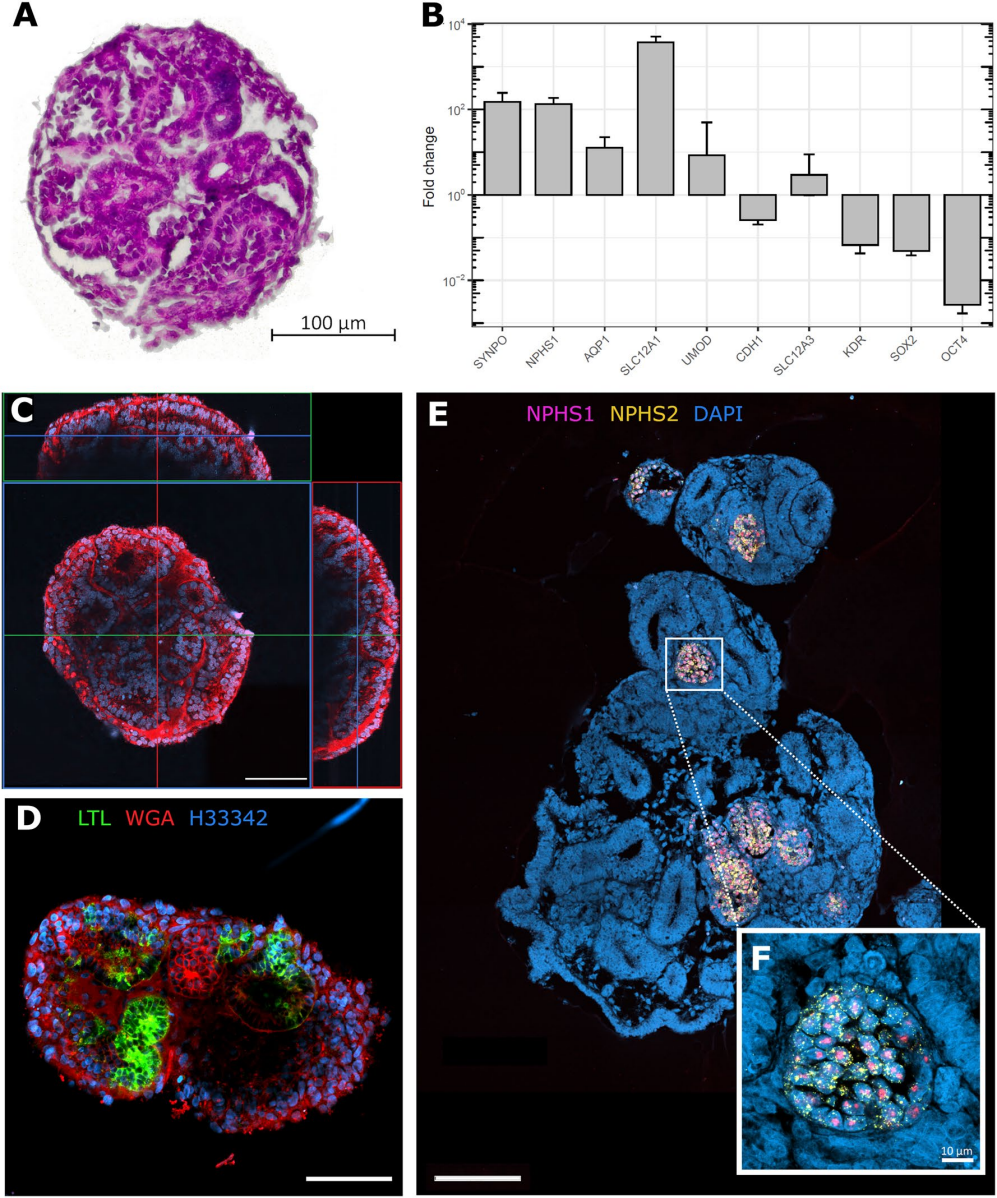
(**A**) H&E stain of a renal organoid. 17-day-old renal organoid; cell nuclei in dark purple, extracellular matrix, and cytoplasm in pink. The organoid resembles tubular epithelial structures similar to in vivo nephrons. Epithelial cells are demarcated by a basement membrane (light pink color) and arranged to form a lumen on their apical side. (**B**) Bar graphs showing fold change for expression of kidney marker genes and hiPSC marker genes (*SOX2, OCT4*) in organoids relative to undifferentiated IMR90 hiPSC determined with qPCR. Data are mean of 3 biological replicates (2 replicates for *SOX2*), with 3 technical replicates for each analyzed gene. Displayed are mean and s.e.m. (**C**) Orthogonal projection of two-photon excitation microscopy analysis of 13-day-old whole-mounted organoid. Displayed are frontal XY, sagittal YZ, and transversal XZ directions. For z-stacks, the sample was imaged from the dish bottom up to 140 µm in z-direction with a slice thickness of 1 µm. The renal organoid showed tubular structures recognizable by circularly arranged nuclei and membranous structures, stained by Hoechst 33342 and WGA TMR, respectively. A lumen within the tubules is evident. (**D**) Organoid with LTL ^+ ^proximal tubules. (**E**) Confocal images of detection of *NPHS1* and *NPHS2* mRNA with RNAscope in an FFPE d 14 renal organoid. Focus-like distribution of hybridization signals for *NPHS1* and *NPHS2*. 20×magnification. (**F**) High-resolution detail of fluorescence signal distribution for *NPHS1* and *NPHS2*. Strong nuclear hybridization signal for *NPHS1* and fine dot-like and evenly distributed signals for *NPHS2*. 63 × magnification with Airyscan 2. Scale bars: 100 µm (**A**,**C**,**D**,**E**), 10 μm (**F**). *WGA* Wheat germ agglutinin, *TMR* Tetramethylrhodamine, *LTL* *Lotus* *tetragonolobus* lectin, *FFPE* Formalin-fixed paraffin-embedded.

In the hematoxylin and eosin (H&E) staining 17-day-old renal organoids show recognizable renal tubular structures with a lumen (Fig. 1A). The epithelial cells of the tubular structures are clearly demarcated by a basement membrane. Two-photon excitation microscopy analysis of 13-day-old renal organoids also showed renal tubules within the organoid, recognizable by circularly arranged nuclei and membranous structures, which stained positive for wheat germ agglutinin (WGA), a lectin that binds to α-*N*-acetyl-glucosamine and sialic acid in the cell membrane (Fig. 1C)^46^. A lumen within the tubules was also evident. The organoids showed enhanced expression of marker genes for podocytes (*SYNPO, NPHS1*), proximal tubules (*AQP1*), the thick ascending limb of the loop of Henle (*SLC12A1, UMOD*), and distal convoluted tubules, suggesting the formation of nephron structures, while hiPSC marker gene expression (*SOX2, OCT4*) was reduced (Fig. 1B)^47–50^.

The presence of proximal tubules was verified by positive staining for *Lotus tetragonolobus* lectin (LTL) (Fig. 1D)^7,51^. RNA fluorescence in situ hybridization also confirmed the expression of the podocyte marker genes *NPHS1* and *NPHS*2 in 14-day-old renal organoids. Signals for both *NPHS1* and *NPHS2* were co-localized in circular structures distinct from renal tubules (Fig. 1E). In high-resolution detail imaging of the circular foci, strong nuclear hybridization signals for *NPHS1* along with fine dot-like and evenly distributed signals for *NPHS2* could be detected (Fig. 1F). Their co-localization strongly suggests the presence of podocytes in glomerular-like structures in the organoids.

### Viability assessment after DOX treatment for 48 h

**Figure 2:**
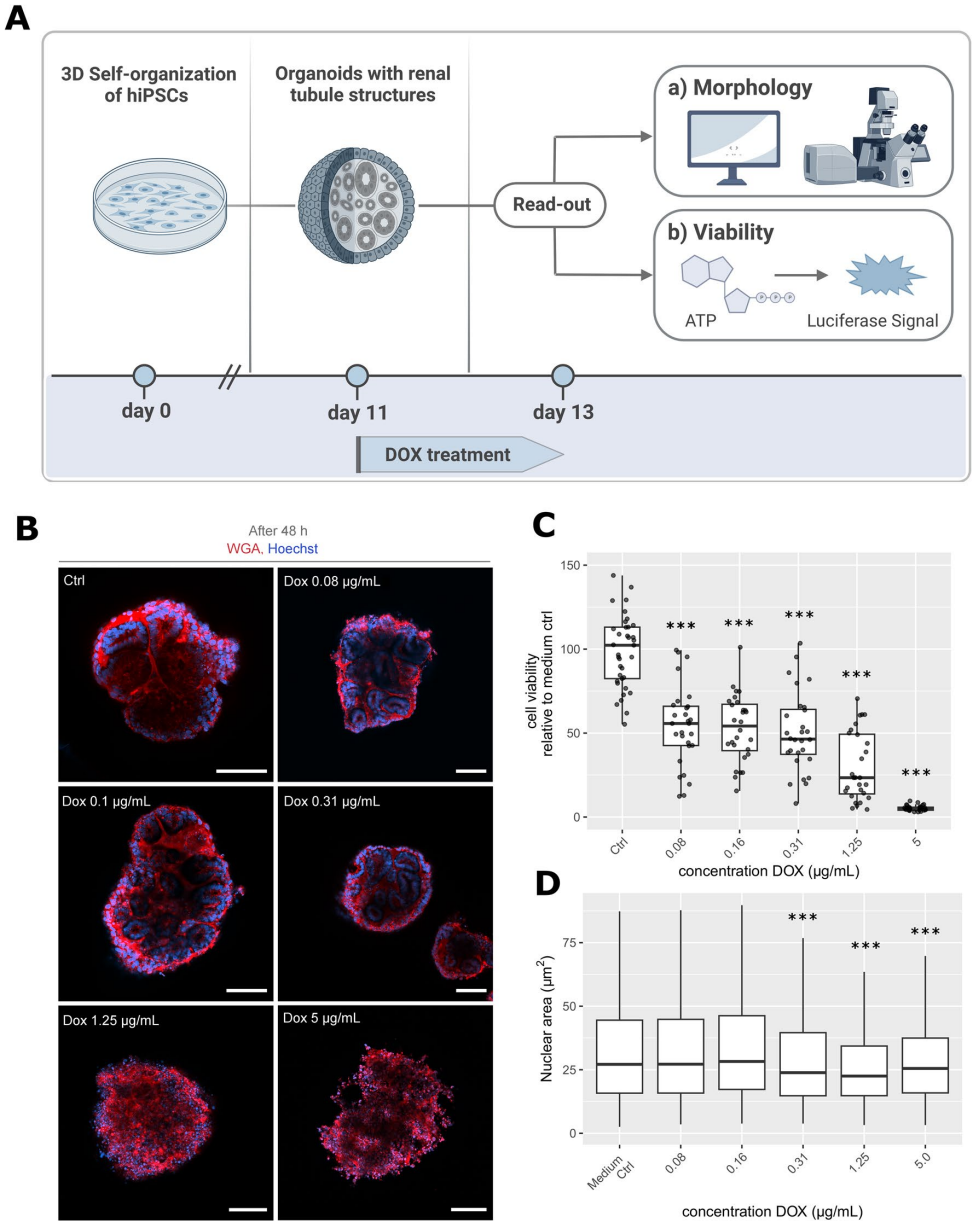
(**A**) Schematic illustration of the course DOX toxicity assay. HiPSCs were seeded in Stage-I medium at day 0. EBs formed, differentiated, and showed renal tubule structures as kidney organoids. By day 11, organoids were singled into a 96-well plate and treated with DOX (0.08–5 µg/mL) and medium controls for 48 h. On day 13, organoids underwent either a histological (a) or a biochemical (b) analysis. (a) DOX-treated organoids and medium control whole-mounted organoids were fluorescently stained as described and morphology was assessed with confocal imaging. (b) Cell viability of DOX-treated organoids was assessed with a luciferase-ATP assay. (**B**) Confocal imaging of DOX-treated renal organoids after 48 h. Whole-mount staining of organoids with Hoechst 33342 (blue, cell nuclei) and WGA 633 (red, cell membrane) enabled visualization of DOX-induced toxicity in organoids. Structural analysis showed visible tubular damage and loss of membrane integrity at higher drug concentrations (1.25–5 µg/mL) compared to untreated controls, which is less observable for lower concentrations. Scale bar: 100 µm. (**C**) Organoid cell viability of DOX-treated kidney organoids and medium control in % (relative to untreated medium control). Renal organoids were treated with DOX concentrations ranging from 5 to 0.08 µg/mL for 48 h. Cell viability as an inverted measure of DOX toxicity was determined with an ATP-luciferase assay. Organoids were lysed, and ATP concentrations were measured luminometrically. High ATP levels equal high metabolic activity of cells, hence high viability. Treated organoids showed significantly lower cell viability compared to the medium control. The viability decreased with increasing DOX concentrations, with the lowest viability (5%) corresponding to the highest DOX concentration (5 µg/mL). Data is plotted as boxplot with whiskers and data points depicted as dots using the ggplot2 v3.4.1 package in R v3.6.3. *p*-values were calculated by one-way ANOVA (*p* < 2*10^−16^) and post-hoc Tukey multiple comparison of means, *** represents statistical significance versus medium control with *p* < 1*10^−7^, n = 4 independent experiments. (**D**) Measurement of nuclear area of DOX-treated organoids. *p*-values were calculated by one-way ANOVA (*p* < 2*10^−16^) and post-hoc Tukey multiple comparison of means; *** represents statistical significance versus medium control with *p* < 1*10^−7^, n = 4 independent experiments. *DOX* Doxorubicin, *HiPSCs* Human induced pluripotent stem cells, *EBs* Embryoid bodies, *ATP* Adenosine triphosphate, *Ctrl* Medium control, *WGA* Wheat germ agglutinin.

Renal organoids were treated with DOX concentrations ranging from 5 to 0.08 µg/mL for 48 h. Organoid viability as an inverted measure of DOX-induced toxicity was assessed through an ATP-luciferase assay (see Fig. 2A). All luminescence values were divided by the organoid’s respective volume and normalized to the mean medium control value. Overall, treated organoids showed significantly lower ATP levels and viability than the medium control (Fig. 2C). The decrease in viability was concentration-dependent, with the lowest cell viability of around 5% corresponding to the highest DOX concentration of 5 µg/mL. The viability for organoids treated with concentrations of 0.08, 0.16, and 0.31 µg/mL did not differ visibly from one another, ranging between 54 and 50%, respectively.

### Imaging of DOX-treated renal organoids

Structural analysis showed visible tubular disintegration and loss of membrane integrity at concentrations of 1.25 and 5 µg/mL compared to untreated controls, correlating with the concentration-dependent decrease in cell viability (Fig. 2B). These histological changes were not visually detectable at lower concentrations of 0.08–0.31 µg/mL, where tubular structures were clearly visible and cells were demarcated by intact cell membranes. Further, the area of the nuclei of the imaged organoids was measured with quPath. It was observed that the mean nuclear area decreases as DOX concentration rises, suggesting that the cells' nuclei fragment more severely at higher doses as a sign of cell death (Fig. 2D)^52^.

## Discussion

DOX, a chemotherapeutic agent, has been proven to cause disintegration of renal tissue in rodent models as well as podocyte injury and an overall occurrence of dose-dependent apoptosis in organoid models^6,7,43–45^.

Within this study, the DOX treatment of renal organoids for 48 h showed a decrease of organoid viability in both the biochemical assessment as well as optically. Organoids treated with concentrations of 1.25 and 5 µg/mL, which correspond to peak plasma concentrations that are reached during cancer treatment, presented with the lowest viability and visible tubular disintegration as well as a loss of cellular boundaries, potentially as a result of increased oxidative stress^42,53^. Any morphological effect of the drug treatment at lower doses could not be identified by visual inspection of the sample images. The morphological appearance was then further examined by measuring the mean nuclear area as an indicator of cell health. We showed, that images of organoids treated with 5 µg/mL and 1.25 µg/mL exhibited a significantly smaller nuclear area, which may be attributed to an increase in nuclear fragmentation^52^. We, therefore, presume that the organoids treated with higher doses exhibit proportionately greater levels of cell deterioration. This is consistent with the measurements of cell viability. Furthermore, not all specimens could be analyzed due to the loss of some organoids during the staining procedure and time restrictions in generating the microscopy images. In general, an automated assessment of morphological markers and a greater number of samples can result in a read-out that is more objective and reproducible. For instance, pattern recognition used in computer-assisted approaches like neural networks might identify morphological changes brought on by treatment. It should be emphasized, however, that appearances may vary from batch to batch, therefore a high number of replicates to increase reproducibility is of great importance. Nevertheless, a tendency for a dose-dependent morphological change of renal organoids upon DOX treatment is also evident in this experimental setup.

However, the assay does not allow any further conclusions on the type of toxicity. To explore specific nephrotoxic effects, the expression of *KIM*-*1* as a marker for renal injury may be studied by immunostaining and qPCR analysis alongside measuring organoid viability and morphological appearance after treatment^54^. Similar studies were done by Morizane and colleagues, who found *KIM-1* expression to be highly upregulated in proximal tubules of renal organoids following gentamicin treatment^54^. Although our morphological data demonstrate that the toxic effect of DOX likely affects the entire organoid structure by showing a loss of cellular boundaries and a decrease in nuclear area, the organoids do contain proliferative embryonic nephrons, which can be targeted by DOX’ anti-proliferative properties^50,55^. Therefore, an additional anti-proliferative effect of DOX in this setup cannot be ruled out completely^56,57^.

Notably, as is the case with the majority of in vitro settings, the toxic effect of DOX in this experiment may differ from its physiological effect. Due to the kidney’s filtering function, the glomerulus and proximal tubule would typically be exposed to the highest drug concentrations. However, because the organoids are grown in suspension culture in this setup, each organoid component is equally exposed to the drug. This may also suggest that in our assay DOX has a more pervasive, generalized toxic effect on the renal organoids that would affect all cell types, rather than only podocytes^58^. In a study conducted by Kumar et al*.* it was shown that similar DOX concentrations dose-dependently reduced the expression of not only podocyte marker genes but all of the examined nephron segment markers, and therefore also affected other nephron parts^7^. Future research based on our protocol may be combined with RNA fluorescence in situ hybridization to investigate the potential effects of DOX on podocytes, in a manner similar to that of Zoja and colleagues who observed a loss of *NPHS1* following DOX treatment^59^.

Although renal organoids have demonstrated their capacity to simulate drug-induced toxicity, the way the experiment was designed may have had an impact on the viability measurements. Because the organoids are not filtered out by size during their differentiation, the organoids used in the toxicity assay varied in diameter. By normalizing the viability measurement to the organoids' individual volumes, we excluded the dependence of viability measurement on cellular mass. Since the organoids are not perfectly elliptical, the measurement of the radius is subjective, and the volumes of the organoids approximated from it are rather estimates. In upscaled approaches, the organoids could be filtered by size beforehand so that all samples consist of approximately the same number of cells. In an alternative attempt to measure cell viability of organoids, Susa and colleagues generated ATP reporter organoids to fluorescently detect the ATP/ADP ratio to allow for live toxicity monitoring^60^.

However, we demonstrated that our established experimental framework for drug toxicity assessment in renal organoids may be utilized in the early stages of research as a valuable addition to current approaches like animal models or 2D cell culture systems. In contrast to other approaches, we provide a straightforward setup that may allow for easier incorporation of renal organoid models to screen for compound toxicity.

## Methods

Cells and organoids were cultured under standard conditions (37 °C, 5% CO_2_) in a CO_2_ incubator and handled under sterile conditions under a biosafety cabinet.

### Generation and culture of hiPSC-derived renal organoids

Renal organoids were generated in suspension culture after the method published by Przepiorski et al*.*^50^. IMR90-derived hiPSC which were used for organoid generation, were kindly provided by Prof. Kurtz from the BCRT (Berlin, Germany). The hiPSC lines are fully consented for research use and quality controlled. HiPSCs were cultured in DEF-CS medium (Cellartis). Cells were allowed to expand until 80–90% confluency in 6-well cell culture plates. HiPSCs were washed once with pre-warmed DPBS and incubated with 1 mL Dispase (1 U/mL, Stemcell Technologies) at room temperature (RT) for 2 min or until cells started to detach. Dispase was removed, and cells were washed twice with DPBS. Stage I medium containing 1X Stemdiff APEL 2 (Stemcell Technologies), 10 µM CHIR99021 (Merck), 5% Protein-Free Hybridoma Medium II (1X) with L-Glutamine (ThermoFisher Scientific), 0.1X Insulin-Transferrin-Selenium-X (ThermoFisher Scientific) and 1X Y-27632 (Stemcell Technologies) was added, and cells were detached with a cell lifter and gently resuspended twice with a 1000 µL pipette tip to break up larger cell aggregates. The cell suspension was divided equally among the wells of a 6-well ultra-low attachment (ULA) cell culture plate—one well at 80% confluency was divided onto two ULA plate wells. Cells were allowed to self-assemble into spherical embryoid bodies (EBs). After 2 days, a half medium change was carried out with 1 mL Stage I medium per well. On day 3, EBs were harvested with a 5 mL serological pipette, transferred to a 15 mL conical centrifuge tube, and centrifuged at 300×*g* for 30 s. Medium was removed, EBs were carefully resuspended in Stage II medium, containing 1X DMEM (high glucose, GlutaMAX Supplement, ThermoFisher Scientific), 15% KnockOut Serum Replacement (ThermoFisher Scientific), 1% MEM NEAA (ThermoFisher Scientific) 1% Penicillin/Streptomycin, 1% HEPES (1 M) and 0.05% Poly-Vinyl-Alcohol (5%, Sigma) using a 5 mL serological pipette and returned to the same wells of the ULA plate. Half medium changes with 1 mL Stage II medium were carried out every second day. When the organoids displayed visible formation of renal tubular structures after 7–8 days, they were transferred onto an orbital shaker at 90 rounds per minute in the incubator and cultured up to day 14.

### Real time-qPCR analysis

Organoid mRNA was isolated with the NucleoSpin RNA plus kit (Macherey–Nagel) and was converted to cDNA using the High Capacity cDNA Reverse Transcription Kit (Applied Biosystems). Quantitative reverse transcription PCR (qPCR) analyses were performed with the Luminaris HiGreen qPCR Master Mix (ThermoFisher Scientific) by a StepOnePlus™ Real-Time PCR System (Applied Biosystems) or with the SsoAdvanced Universal SYBR Green Supermix (Bior-Rad) by a CFX Opus 384 Real-Time PCR System (Bio-Rad). All data were first normalized to *TBP *and *EMC7* and then normalized to undifferentiated IMR90 control samples using the deltadelta Ct method. Primer sequences can be found in Table 1 in the supplementary information.

### Fluorescent staining of whole-mount organoids

Organoids were stained using a whole-mount approach and imaged by laser scanning microscopy. This allowed deeper layers of the organoid to be imaged without prior tissue clearing. Organoids were collected and transferred into 1.5 mL reaction tubes using a cut-off 200 µL pipette tip, making sure not to damage the organoids. They were allowed to settle by gravity before careful aspiration of Stage II medium. All wash steps were carried out with DPBS at RT for 20 min. Organoids were gently resuspended by dispensing DPBS on the side of the 1.5 mL reaction tube. Organoids were washed once and immediately fixed in ice-cold 4% Roti Histofix for at least 24 h at 4 °C or 30 min at RT. Subsequently, organoids were washed three times and permeabilized with 0.5% Triton-X-100 in DPBS for 24 h at 4 °C, followed by another three wash steps. Organoids were dyed with 4 µg/mL Hoechst 33342 (20 mM) (ThermoFisher Scientific) and 30 µg/mL WGA AF 633 conjugate (1 mg/mL) (Invitrogen) or WGA TMR (for two-photon excitation microscopy) for at least 24 h at 4 °C in the dark. Dyes were diluted in DPBS. For Biotinylated *Lotus tetragonolobus* lectin (LTL) (2 mg/mL, BioWorld) and streptavidin AF 635 (2 mg/mL, Invitrogen) whole-mount staining, dyes were prepared in 5% bovine serum albumin in DPBS to a final concentration of 10 µg/mL. Organoids were washed and incubated with biotinylated LTL at 4 °C overnight. On the next day, organoids were washed three times with DPBS for 10 min per wash step. Organoids were incubated with streptavidin AF 635 at 4 °C overnight. The next day, they were washed three times with DPBS and counterstained with 4 µg/mL Hoechst 33342 for 1 h. For microscopical assessment, organoids were washed, carefully resuspended in DPBS, and transferred to a black glass-bottom 96-well microplate (Greiner).

### RNA fluorescent in situ hybridization

For spatial gene expression analysis, we used the RNAscope assay. A detailed method description can be found in the supplementary information. In short, organoids were fixed and sectioned using standard histological procedures. We analyzed the spatial distribution of gene expression for two target genes, *NPHS1* and *NPHS2.* The RNAscope Multiplex Fluorescent v2 Assay was executed according to the manufacturer protocol. The slides were imaged confocally.

### Doxorubicin toxicity assay

Renal organoids were cultured for 11 days and showed visible tubular structures. Organoids were sorted visually by size. Only organoids ranging between 500 and 800 µm were singled and transferred into 0.5 mL reaction tubes using cut-off 200 µL pipette tips. After organoids settled by gravity, they were carefully washed once with DPBS. DPBS was replaced by five different concentrations of DOX (5–0.08 µg/mL) in Stage-II medium and a medium control with only Stage-II medium. Organoids were transferred into a 96-well plate using cut-off 200 µL pipette tips and cultured under standard conditions for 48 h.

### Imaging

For microscopical assessment, whole-mount fluorescence staining was performed accordingly. For imaging of the DOX/medium treated organoids, we aimed at imaging at least three organoids per treatment with confocal laser scanning microscopy (LSM 980, Zeiss), equipped with the objectives 10x (EC Plan-Neofluar 10x/0.30 M27-Air), 20x (Plan-Apochromat 20x/0.8 M27-Air), 40x (LD LCI Plan-Apochromat 40x/1.2 Imm Korr DIC M27-Water) and 63x (C-Apochromat 63x/1.20 W Korr UV VIS IR-Water). Microscope settings (exposure time, pinhole width, laser power, magnification) were adapted to the respective samples to ensure optimal signal detection in every sample. Generally, the pinhole width was kept at 1 airy unit, laser power was kept < 5%, and gain was not adjusted. Images were processed using the ZEN 3.4 software (Zeiss). Black, white, and gamma values for each image were adjusted individually to ensure the best possible visibility of the respective target structures. Focal planes with a distance of 30–50 µm from the bottom of the sample carrier were imaged.

Two-photon excitation microscopy was performed on a Zeiss LSM 710 NLO with two non-descanned detectors using a Ti:Sa laser and 40 × objective (C Apochromat 40x/1.20 W Korr M27). For z-stacks, the sample was imaged from the dish bottom up to 140 µm in z-direction with a slice thickness of 1 µm.

For RNAscope, images were acquired with the Zeiss LSM980 using the multitrack option, so each fluorophore (see Table 2, supplementary information) was detected separately in a single track to avoid crosstalk between channels.

### Detection of nuclear area

To measure the nuclear area, we used QuPath v0.4.2 and its built-in cell detection tool^61^. We optimized the parameters to include an optimal detection of larger and smaller nuclear fragments. The following parameters with the detection channel for Hoechst 33342 were used: requested pixel size 0.5 µm, background radius 8 µm, median filter radius 0 µm, sigma 2 µm, minimum area 5 µm^2^, maximum area 400 µm^2^, threshold 200 with the option split by shape, cell expansion 5 µm and included cell nucleus, score compartment was set to nucleus: H33342-T2-max with single threshold checked. For each concentration or medium control, 7 images were analyzed.

### Determination of cell viability

Cell viability after DOX treatment was determined by a luciferase adenosine triphosphate (ATP) assay (CellTiter-Glo 3D Cell Reagent, Promega) as an inverted measure of DOX toxicity. The ATP quantification was used as a marker for metabolically active cells^62^. Before the ATP quantification, microscopic images of each organoid were taken. The short (y-axis) and long (x-axis) radii of each organoid were measured using ImageJ. The modified equation for a rotation ellipsoid, which assumes that the y and z axes are equal in length and are thus represented by *a*^2^ in the equation, was used to determine the organoid volumes:

1 $${\text{V}} = \frac{{4 {\uppi }}}{{3 a^{2} b}}$$

where V: organoid volume; a: short organoid radius; b: long organoid radius.

The CellTiter-Glo reagent was added to each well (volume 1:1). The plate was shaken linearly at 1440 rpm for 5 min and incubated for 25 min at RT. During the incubation period, organoid cells were lysed, and ATP was released. The luminescence was measured in a luminometer (Tecan) with an integration time of 1000 ms. The luminescence signal of each organoid was divided by their respective volume. All values were normalized to the mean value of the medium control, which was treated equally. Four independent experiments were performed, each assessing 5 different DOX treatment concentrations and with each an associated medium control. Per treatment concentration/medium control, 7 organoids were measured. For the experiments 1–3, we included 10 organoid measurements per medium control. A total of 177 organoids were analyzed.

### Statistical analysis

Data was analyzed using the ggplot2 v3.4.1 package in R v3.6.3. *p*-values were calculated by one-way ANOVA (*p* < 2*10^−16^) and post-hoc Tukey multiple comparison of means where *** represents statistical significance versus medium control with *p* < 1*10^−7^ of n = 4 independent experiments.

## Supplementary Information

Supplementary Information.

## Data Availability

The datasets generated and analyzed during the current study are available from the corresponding author on reasonable request.
