# Supplementary material for: A proof-of-concept assay for quantitative and optical assessment of drug-induced toxicity in renal organoids
Source: Sci Rep. 2023 Apr 15;13:6167. doi: 10.1038/s41598-023-33110-5 (PMC10105743; doi:10.1038/s41598-023-33110-5)
Supplement: Supplementary file 1 — Supplementary Information. [file 41598_2023_33110_MOESM1_ESM.pdf]

Supplementary Information

A proof-of-concept assay for quantitative and optical assessment of drug-induced toxicity in renal organoids

Jasmin Dilz[1*], Isabel Auge[1], Kathrin Groeneveld[1], Stefanie Reuter[2], Ralf Mrowka[1,2*]

Supplementary Methods

Real time-qPCR

Table 1: qPCR primers used in this study

	Forward	Reverse
**SYNPO**	CCGGAGGCTTTGAGAACCAA	CCTTCTCCGTGAGGCTAGTG
**NPHS1**	TCATGTGGTACAAGGACTCGC	TCCCAGATTTCTCCACGCTG
**KDR**	ACCGGCTGAAGCTAGGTAAG	CGATGCTCACTGTGTGTTGC
**CDH1**	GAGGACCAGGACTTTGACTT	AGATACCGGGGGACACTCA
**AQP1**	AAGCTCTTCTGGAGGGCAG	CACCTTCACGTTGTCCTGGACCG
**SLC12A3**	CACCAAGAGGTTTGAGGACAT	GACAGTGGCCTCATCCTTGA
**SLC12A1**	TTTGGAGCTGTTTTGTGCTG	ATGGGTCCCCCTGTTAAGAC
**UMOD**	AAGAGTCTGGGCTTCGACAA	GCTGTAAGTGGCATGGGTTT
**OCT4**	CCTGAAGCAGAAGAGGATCACC	AAAGCGGCAGATGGTCGTTTGG
**SOX2**	GCTACAGCATGATGCAGGACCA	TCTGCGAGCTGGTCATGGAGTT
**EMC7**	TCCTGACATGAGACGGGAAATG	CGCTGCTAGATTTGCCAGATG
**TBP**	GGCGTGTGAAGATAACCCAAGG	CGCTGGAACTCGTCTCACT

RNA fluorescent in situ hybridization

Spatial gene expression was detected with RNA fluorescent in situ hybridization using the RNAscope Multiplex Fluorescent v2 Assay. The target genes *NPHS1* and *NPHS2* were assigned to specific channels (Table 2).The 14-day-old renal organoids were harvested using a cut-off 200 µL pipette tip and transferred to 0.5 mL reaction tubes to sediment. They were carefully washed once with DPBS and immediately fixed with ice-cold 4 % Roti Histofix for at least 24 h at 4 °C.

Table 2: Description of probes for respective target genes.

**Target gene**	**Nphs1**	**Nphs2**
**Channel**	2	1
**Species**	Human	Human
**Entrez Gene ID**	4868	7827
**Accession No**	NM_004646.3	NM_014625.3
**Target Region (Base pairs)**	240 - 1179	268 - 1172
**No. Of pairs**	20	20
**Assigned fluorescent dye**	Opal™ 570	Opal™ 690
**excitation/emission maxima (nm)**	550 / 570	676 / 694

Paraffin sectioning and deparaffinization

For paraffin sectioning, organoids were washed twice with DPBS and transferred to a microscopic slide. Leftover DPBS surrounding the organoids was removed carefully with a tissue. 2 % agarose was heated until liquid and overlaid on the organoids. Agarose was let to solidify at RT and transferred into a biopsy tissue cassette followed by dehydration (Table 3), using fresh solutions for each step. Organoids in agarose were transferred to an embedding cassette and embedded with paraffin using the TES 99.250 system. The paraffin block was let to solidify at RT before storing it at 4 °C for at least 3 days. Paraffin-embedded organoids were sectioned using a RM2155 Rotary Microtome at 4 - 6 µm thickness. Sections were straightened out in a 40 – 45 °C water bath, transferred onto SuperFrost Plus™ adhesion slides, and dried at RT. Dried slides were baked in the dry HybEZ™ II Hybridization System oven in "bake mode" at 60 °C for 1 h. Embedded organoids were deparaffinized at RT on an orbital shaker at 70 rpm according to Table 4 and dried at RT.

Table 3: Steps for organoid dehydration

Step	Solution	Incubation time
1	70 % Ethanol (denatured)	1 h
2	96 % Ethanol (denatured) (I)	1 h
3	96 % Ethanol (denatured) (II)	1 h
4	Isopropyl Alcohol (I)	1.5 h
5	Isopropyl Alcohol (II)	1.5 h
6	Xylol (I)	1.5 h
7	Xylol (II)	1.5 h
8	Paraffin at 52 - 54°C (I)	Over night
9	Paraffin at 52 - 54°C (III)	1.5 h
10	Paraffin at 52 - 54°C (III)	1.5 h

Table 4: Steps for organoid deparaffinization

Step	Solution	Incubation time
1	Neoclear (I)	10 min
2	Neoclear (II)	10 min
3	100 % Ethanol (I)	2 min
4	100 % Ethanol (II)	2 min

Sample pre-treatment

The pre-treatment steps were executed according to the manufacturer protocol for formalin-fixed paraffin-embedded tissue (FFPE) (ACD, document number 323100-USM/Rev Date: 02272019). Target retrieval was performed in a steam cooker at approximately 100 °C for 15 min. All following wash steps of the protocol were carried out on an orbital shaker at 70 rpm, either in staining jars (Hellendahl) or in the RNAscope EZ-Batch wash tray, where indicated. Wash buffer in the staining jars was replaced after every wash step.

RNA *in situ* hybridization using RNAscope

The RNAscope Multiplex Fluorescent v2 Assay was executed according to the manufacturer protocol listed above for the sample pre-treatment. The two target probes for *NPHS1* and *NPHS2* detection were assigned to their respective channels and fluorescent dyes according to Table 2. For channel development and amplification with fluorescent dyes, the staining jars and the wash tray were covered in aluminum foil to protect the samples from light. Sample

slides were mounted with ProLong Gold Antifade mounting medium and stored in a dark slide box at 4 °C. The slides were imaged with the inverted confocal laser scanning microscope (LSM) 980 from Zeiss, as mentioned.

Table 5: Summary of viability measurements of the DOX toxicity assays.

	Control	0,08 µg/mL	0,16 µg/mL	0,31 µg/mL	1,25 µg/mL	5 µg/mL
**Number of values**	37	28	28	28	28	28
**Minimum**	55,23	12,34	15,58	8,07	4,52	3,03
**25% Percentile**	81,63	42,48	38,08	35,49	12,9	4,05
**Median**	102,3	55,71	54,19	46,42	23,43	5,09
**75% Percentile**	113,6	66,39	67,97	64,95	49,77	6,05
**Maximum**	164,5	99,4	101,1	103,5	70,52	9,6
**Mean**	100	54,3	52,8	50,3	29,7	5,31
**Std. Deviation**	23,91	23,76	19,95	23,62	20,43	1,65
**Std. Error**	3,93	4,49	3,77	4,46	3,86	0,31

Table 6: Organoid viability after 48h DOX treatment. Data acquired with 4 separate assays, viability measurements in % relative to untreated medium control.

Assay number	Replicate	Organoid viability (%) with corresponding DOX concentration (µg/mL)				
		**5**	**1.25**	**0.3**	**0.15**	**0.08**
**1**	1	6,15	38,75	49,98	66,76	49,37
	2	4,04	61,01	103,51	71,37	23,72
	3	3,82	60,63	95,33	51,69	99,4
	4	4,24	49,99	85,98	63,47	88,38
	5	4,47	55,48	66,04	77,56	98,13
	6	5,55	23,5	82,04	43,52	71,61
	7	4,09	61,03	50,88	74,97	55,71
**2**	1	5,48	6,71	22,22	35,47	33,27
	2	5,59	14,21	8,07	26,46	24,81
	3	9,60	8,30	19,46	44,49	12,86
	4	8,68	8,44	23,22	26,44	19,51
	5	5,67	11,45	38,37	57,64	42,42
	6	5,47	4,52	60,34	15,58	12,34
	7	6,99	5,17	19,84	23,72	69,00
**3**	1	7,39	49,11	46,69	101,12	64,90
	2	4,03	70,52	38,27	56,69	42,64
	3	4,44	51,91	39,54	63,66	49,47
	4	5,77	25,42	53,49	37,35	66,60
	5	7,54	17,43	65,37	62,38	65,77
	6	6,91	43,86	50,60	69,01	57,90
	7	5,56	34,73	79,72	40,25	57,07
**4**	1	44,24	42,89	46,00	23,44	4,65
	2	60,58	46,06	39,97	12,46	3,28
	3	61,03	74,8	46,14	16,16	3,03
	4	95,39	47,22	63,69	15,49	3,99
	5	55,71	26,83	33,59	23,42	3,08
	6	48,25	68,37	45,39	19,29	4,71
	7	50,31	62,58	34,56	19,30	4,32
